# Supplementary material for: A latent class assessment of healthcare access factors and disparities in breast cancer care timeliness
Source: PLoS Med. 2024 Dec 2;21(12):e1004500. doi: 10.1371/journal.pmed.1004500 (PMC11649116; doi:10.1371/journal.pmed.1004500)
Supplement: S1 Table — ER, estrogen receptor; HER2, human epidermal growth factor receptor 2. (DOCX) [file pmed.1004500.s002.docx]

|  | Overall | Non-Black | Black |
| --- | --- | --- | --- |
|  | N=2,998 | N=1,503 | N=1,495 |
| Age |  |  |  |
| <50 years | 1,492 (50%) | 751 (50%) | 741 (50%) |
| 50+ years | 1,506 (50%) | 752 (50%) | 754 (50%) |
| Tumor Size |  |  |  |
| <2 cm | 1,538 (51%) | 878 (59%) | 660 (44%) |
| 2-5 cm | 1,081 (36%) | 481 (32%) | 600 (40%) |
| 5+ cm | 368 (12%) | 138 (9.2%) | 230 (15%) |
| Tumor Stage |  |  |  |
| I | 1,225 (41%) | 713 (47%) | 512 (34%) |
| II | 1,225 (41%) | 567 (38%) | 658 (44%) |
| III | 436 (15%) | 186 (12%) | 250 (17%) |
| IV | 109 (3.6%) | 36 (2.4%) | 73 (4.9%) |
| ER Status |  |  |  |
| Negative | 895 (30%) | 326 (22%) | 569 (38%) |
| Positive | 2,094 (70%) | 1,173 (78%) | 921 (62%) |
| HER2 Status |  |  |  |
| Negative | 2,535 (85%) | 1,287 (86%) | 1,248 (84%) |
| Positive | 451 (15%) | 211 (14%) | 240 (16%) |
