# Supplementary material for: A latent class assessment of healthcare access factors and disparities in breast cancer care timeliness
Source: PLoS Med. 2024 Dec 2;21(12):e1004500. doi: 10.1371/journal.pmed.1004500 (PMC11649116; doi:10.1371/journal.pmed.1004500)
Supplement: S2 Table — Counts and frequencies of variables used to form latent classes. Travel times were based on estimated driving distance between patient residence and healthcare facilities. Breast cancer screening adherence was defined among patients age >45 based on receipt of at least 1 mammogram every 2 years. (DOCX) [file pmed.1004500.s003.docx]

|  | Overall | Non-Black | Black |
| --- | --- | --- | --- |
| **SES** |  |  |  |
| Income |  |  |  |
| <$15,000 | 461 (16%) | 105 (7.4%) | 356 (25%) |
| 20,000$ to $50,000 | 1,083 (38%) | 434 (30%) | 649 (46%) |
| $50,000+ | 1,295 (46%) | 885 (62%) | 410 (29%) |
| Education |  |  |  |
| <High School | 239 (8.0%) | 70 (4.7%) | 169 (11%) |
| High School | 933 (31%) | 419 (28%) | 514 (34%) |
| Some college | 1,825 (61%) | 1,014 (67%) | 811 (54%) |
| Country of birth |  |  |  |
| USA | 2,841 (95%) | 1,393 (93%) | 1,448 (97%) |
| non-USA | 157 (5.2%) | 110 (7.3%) | 47 (3.1%) |
| Job type |  |  |  |
| Farmer, service worker, other laborer | 597 (20%) | 182 (12%) | 415 (28%) |
| Craftworker, factory worker, Mechanic | 292 (9.9%) | 86 (5.8%) | 206 (14%) |
| Clerical worker, sales, technician | 647 (22%) | 349 (24%) | 298 (20%) |
| Professional, administrator, Executive | 1,413 (48%) | 863 (58%) | 550 (37%) |
| Marital status |  |  |  |
| Not married | 1,299 (43%) | 424 (28%) | 875 (59%) |
| Married | 1,698 (57%) | 1,079 (72%) | 619 (41%) |
| **Care barriers** |  |  |  |
| Insurance |  |  |  |
| Insured | 2,817 (94%) | 1,446 (96%) | 1,371 (92%) |
| Not insured | 179 (6.0%) | 56 (3.7%) | 123 (8.2%) |
| Census tract urbanicity |  |  |  |
| Urban | 2,589 (86%) | 1,301 (87%) | 1,288 (86%) |
| Rural | 409 (14%) | 202 (13%) | 207 (14%) |
| Job loss due to breast cancer diagnosis |  |  |  |
| No | 2,855 (96%) | 1,447 (97%) | 1,408 (95%) |
| Yes | 118 (4%) | 46 (3%) | 72 (5%) |
| Self-reported financial barriers |  |  |  |
| No | 2,479 (83%) | 1,347 (90%) | 1,132 (76%) |
| Yes | 518 (17%) | 156 (10%) | 362 (24%) |
| Self-reported transportation barriers |  |  |  |
| No | 2,835 (95%) | 1,472 (98%) | 1,363 (91%) |
| Yes | 162 (5.4%) | 31 (2.1%) | 131 (8.8%) |
| **Care use** |  |  |  |
| Pre-diagnostic regular care |  |  |  |
| No | 2,602 (87%) | 1,382 (92%) | 1,220 (82%) |
| Yes | 396 (13%) | 121 (8.1%) | 275 (18%) |
| Screening adherent |  |  |  |
| Yes | 1,375 (67%) | 745 (71%) | 630 (63%) |
| No | 683 (33%) | 307 (29%) | 376 (37%) |
| Mode of detection |  |  |  |
| Lump detected | 1,187 (43%) | 670 (49%) | 517 (38%) |
| Routine mammogram | 1,548 (57%) | 702 (51%) | 846 (62%) |
| Driving time to diagnosis |  |  |  |
| <30 mins | 2,229 (74%) | 1,043 (69%) | 1,186 (79%) |
| 30-60 mins | 620 (21%) | 378 (25%) | 242 (16%) |
| 60+ mins | 146 (5%) | 82 (6%) | 64 (4%) |
| Driving time to surgery |  |  |  |
| <30 mins | 2,069 (69%) | 953 (63%) | 1,116 (75%) |
| 30-60 mins | 673 (23%) | 405 (27%) | 268 (18%) |
| 60+ mins | 248 (8%) | 144 (10%) | 104 (7%) |
