# Supplementary material for: A latent class assessment of healthcare access factors and disparities in breast cancer care timeliness
Source: PLoS Med. 2024 Dec 2;21(12):e1004500. doi: 10.1371/journal.pmed.1004500 (PMC11649116; doi:10.1371/journal.pmed.1004500)
Supplement: S3 Table — Cell values correspond to relative frequency differences (RFDs) and 95% confidence intervals. The main analysis column represents the CBCS population without exclusion criteria. The black and white patients only column (N = 2,916) represents a sensitivity analysis with Asian, American Indian, and other races excluded. The Stage I–III column (N = 2,886) represents a sensitivity analysis with stage IV patients excluded (not shown for prolonged treatment and OncotypeDx, as these analyses already excluded stage IV patients in the main analysis). RFDs for each outcome are compared between latent class categories, defined for SES (income, education, country of birth, job type, and marital status), care barriers (insurance, urban/rural status, job loss, self-reported financial barriers to care, self-reported transportation barriers to care), and care use (pre-diagnostic regular care, breast cancer screening, mode of initial cancer detection (mammogram vs. noticed lump), and travel (based on estimated driving time) to diagnosis and surgery). (DOCX) [file pmed.1004500.s004.docx]

|  | Main analysis | Black and White Patients only | Stage I-III patients only | Main analysis | Among Black and White Patients only | Stage I-III patients only |
| --- | --- | --- | --- | --- | --- | --- |
|  | Delayed diagnosis | | | Delayed treatment | | |
| **SES** |  |  |  |  |  |  |
| High SES | 0 (ref) |  |  | 0 (ref) |  |  |
| Moderate SES | 6.0 (2.4, 9.5) | 6.0 (2.4, 9.6) | 4.8 (1.4, 8.1) | 1.0 (-3.5, 5.5) | 0.2 (-4.3, 4.8) | 0.4 (-4.2, 5.0) |
| Low SES | 5.5 (2.4, 8.5) | 5.1 (2.0, 8.2) | 4.1 (1.2, 6.9) | 0.1 (-3.9, 4.1) | -0.2 (-4.3, 3.9) | 0.3 (-3.7, 4.4) |
| **Care barriers** |  |  |  |  |  |  |
| Few Barriers | 0 (ref) |  |  | 0 (ref) |  |  |
| More barriers | 6.7 (2.8, 10.7) | 6.3 (2.3, 10.3) | 4.7 (0.9, 8.5) | -2.0 (-6.5, 2.6) | -1.8 (-6.4, 2.8) | -3.1 (-7.7, 1.5) |
| **Care use** |  |  |  |  |  |  |
| Short Travel, High use | 0 (ref) |  |  | 0 (ref) |  |  |
| Short Travel, Low Use | 17.4 (12.6, 22.2) | 17.5 (12.6, 22.4) | 12.8 (8.1, 17.5) | -4.9 (-10.0, 0.2) | -4.5 (-9.7, 0.6) | -5.9 (-11.1, -0.7) |
| Medium Travel | -0.3 (-3.6, 3.1) | -0.5 (-3.9, 2.9) | 0.3 (-3.0, 3.5) | 0.3 (-4.3, 4.8) | 0.2 (-4.5, 4.8) | 0.6 (-4.0, 5.3) |
| Variable Travel | 1.4 (-4.5, 7.3) | 2.0 (-4.1, 8.2) | 0.8 (-4.9, 6.4) | 10.7 (2.7, 18.8) | 10.2 (2.1, 18.4) | 11.0 (2.9, 19.1) |
| Long Travel | 4.3 (-3.3, 11.9) | 5.1 (-2.8, 13.0) | 4.8 (-2.6, 12.3) | 1.1 (-7.5, 9.8) | 2.3 (-6.6, 11.3) | 1.2 (-7.5, 10.0) |
|  |  |  |  |  |  |  |
| **SES** | Prolonged treatment | | | Not OncotypeDx tested | | |
| High SES | 0 (ref) |  |  | 0 (ref) |  |  |
| Moderate SES | 9.7 (4.8, 14.6) | 9.8 (4.8, 14.7) |  | 5.1 (-1.1, 11.3) | 4.9 (-1.4, 11.3) |  |
| Low SES | 10.1 (5.7, 14.5) | 10.2 (5.7, 14.6) |  | 7.1 (1.5, 12.6) | 7.1 (1.4, 12.7) |  |
| **Care barriers** |  |  |  |  |  |  |
| Few Barriers | 0 (ref) |  |  | 0 (ref) |  |  |
| More barriers | 7.3 (2.4, 12.2) | 7.2 (2.3, 12.2) |  | 2.9 (-3.7, 9.5) | 2.7 (-3.9, 9.3) |  |
| **Care use** |  |  |  |  |  |  |
| Short Travel, High use | 0 (ref) |  |  | 0 (ref) |  |  |
| Short Travel, Low Use | 1.9 (-3.7, 7.5) | 1.2 (-4.4, 6.9) |  | 3.7 (-4.4, 11.8) | 3.2 (-4.9, 11.4) |  |
| Medium Travel | -4.9 (-9.9, 0.1) | -4.9 (-10.0, 0.1) | - | -3.0 (-9.2, 3.3) | -3.5 (-9.8, 2.8) |  |
| Variable Travel | 1.7 (-6.9, 10.3) | 0.7 (-8.0, 9.4) |  | -5.3 (-15.4, 4.8) | -5.5 (-16.0, 4.9) |  |
| Long Travel | -3.0 (-12.7, 6.7) | -0.9 (-10.9, 9.1) |  | -1.0 (-14.3, 12.2) | 0.4 (-13.2, 14.0) |  |
| Cell values correspond to Relative Frequency Differences (RFDs) and 95% confidence intervals  The main analysis column represents the CBCS population without exclusion criteria. The Black and White patients only column (N=2,916) represents a sensitivity analysis with Asian, American Indian, and other races excluded. The Stage I-III column (N=2,886) represents a sensitivity analysis with stage IV patients excluded (not shown for prolonged treatment and OncotypeDx, as these analyses already excluded stage IV patients in the main analysis). | | | | | | |
