# Supplementary material for: A latent class assessment of healthcare access factors and disparities in breast cancer care timeliness
Source: PLoS Med. 2024 Dec 2;21(12):e1004500. doi: 10.1371/journal.pmed.1004500 (PMC11649116; doi:10.1371/journal.pmed.1004500)
Supplement: S4 Table — Latent variables were defined for SES (income, education, country of birth, job type, and marital status), care barriers (insurance, urban/rural status, job loss, self-reported financial barriers to care, self-reported transportation barriers to care), and care use (pre-diagnostic regular care, breast cancer screening, mode of initial cancer detection (mammogram vs. noticed lump), and travel (based on estimated driving time) to diagnosis and surgery). (DOCX) [file pmed.1004500.s005.docx]

|  |  |  |
| --- | --- | --- |
| Latent class cross-classification | Frequency | Percent |
| High SES, few barriers, short travel / high use | 693 | 23.1 |
| High SES, few barriers, short travel / low use | 54 | 1.8 |
| High SES, few barriers, medium travel | 203 | 6.8 |
| High SES, few barriers, variable travel | 74 | 2.5 |
| High SES, few barriers, long travel | 36 | 1.2 |
| High SES, more barriers, short travel / high use | 21 | 0.7 |
| High SES, more barriers, short travel / low use | 4 | 0.1 |
| High SES, more barriers, medium travel | 9 | 0.3 |
| High SES, more barriers, variable travel | 3 | 0.1 |
| High SES, more barriers, long travel | 3 | 0.1 |
| Moderate SES, few barriers, short travel / high use | 358 | 11.9 |
| Moderate SES, few barriers, short travel / low use | 69 | 2.3 |
| Moderate SES, few barriers, medium travel | 83 | 2.8 |
| Moderate SES, few barriers, variable travel | 21 | 0.7 |
| Moderate SES, few barriers, long travel | 15 | 0.5 |
| Moderate SES, more barriers, short travel / high use | 82 | 2.7 |
| Moderate SES, more barriers, short travel / low use | 39 | 1.3 |
| Moderate SES, more barriers, medium travel | 35 | 1.2 |
| Moderate SES, more barriers, variable travel | 10 | 0.3 |
| Moderate SES, more barriers, long travel | 14 | 0.5 |
| Low SES, few barriers, short travel / high use | 483 | 16.1 |
| Low SES, few barriers, short travel / low use | 131 | 4.4 |
| Low SES, few barriers, medium travel | 172 | 5.7 |
| Low SES, few barriers, variable travel | 48 | 1.6 |
| Low SES, few barriers, long travel | 38 | 1.3 |
| Low SES, more barriers, short travel / high use | 113 | 3.8 |
| Low SES, more barriers, short travel / low use | 118 | 3.9 |
| Low SES, more barriers, medium travel | 49 | 1.6 |
| Low SES, more barriers, variable travel | 8 | 0.3 |
| Low SES, more barriers, long travel | 12 | 0.4 |
