# Supplementary figures and images for: A latent class assessment of healthcare access factors and disparities in breast cancer care timeliness
Source: PLoS Med. 2024 Dec 2;21(12):e1004500. doi: 10.1371/journal.pmed.1004500 (PMC11649116; doi:10.1371/journal.pmed.1004500)

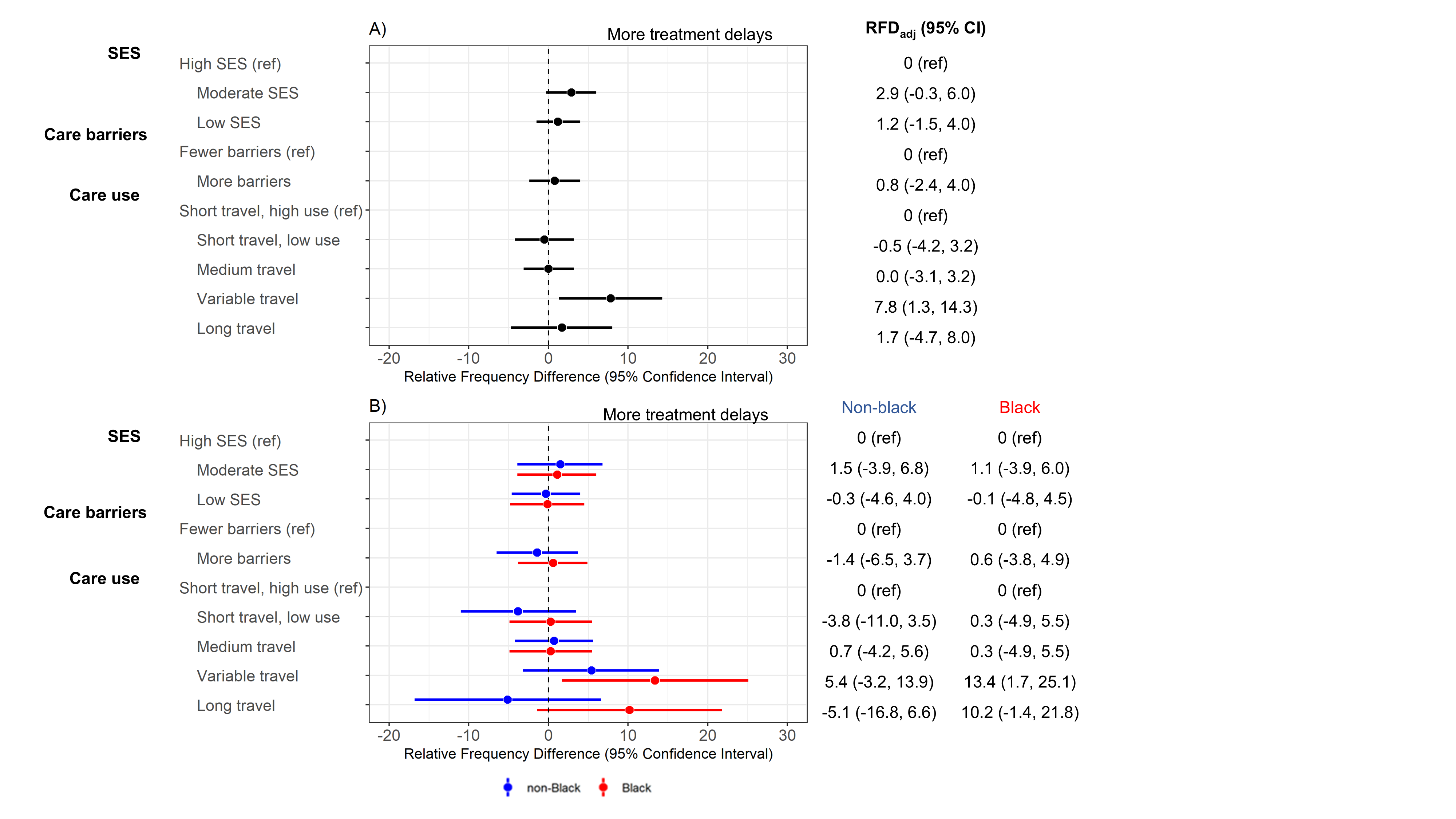

Supplement: S2 Fig — Delayed treatment was assessed in relation to latent class membership defined for latent variables defined for SES (income, education, country of birth, job type, and marital status), care barriers (insurance, urban/rural status, job loss, self-reported financial barriers to care, self-reported transportation barriers to care), and care use (pre-diagnostic regular care, breast cancer screening, mode of initial cancer detection (mammogram vs. noticed lump), and travel (based on estimated driving time) to diagnosis and surgery). Estimates are relative frequency differences (RFDs) and 95% confidence intervals, which compare frequency of delayed treatment for a given latent class with the indicated reference group. Results are presented (A) overall and (B) race-stratified; models are adjusted for age, stage, size, and grade at diagnosis. (TIF) [file pmed.1004500.s007.tif]

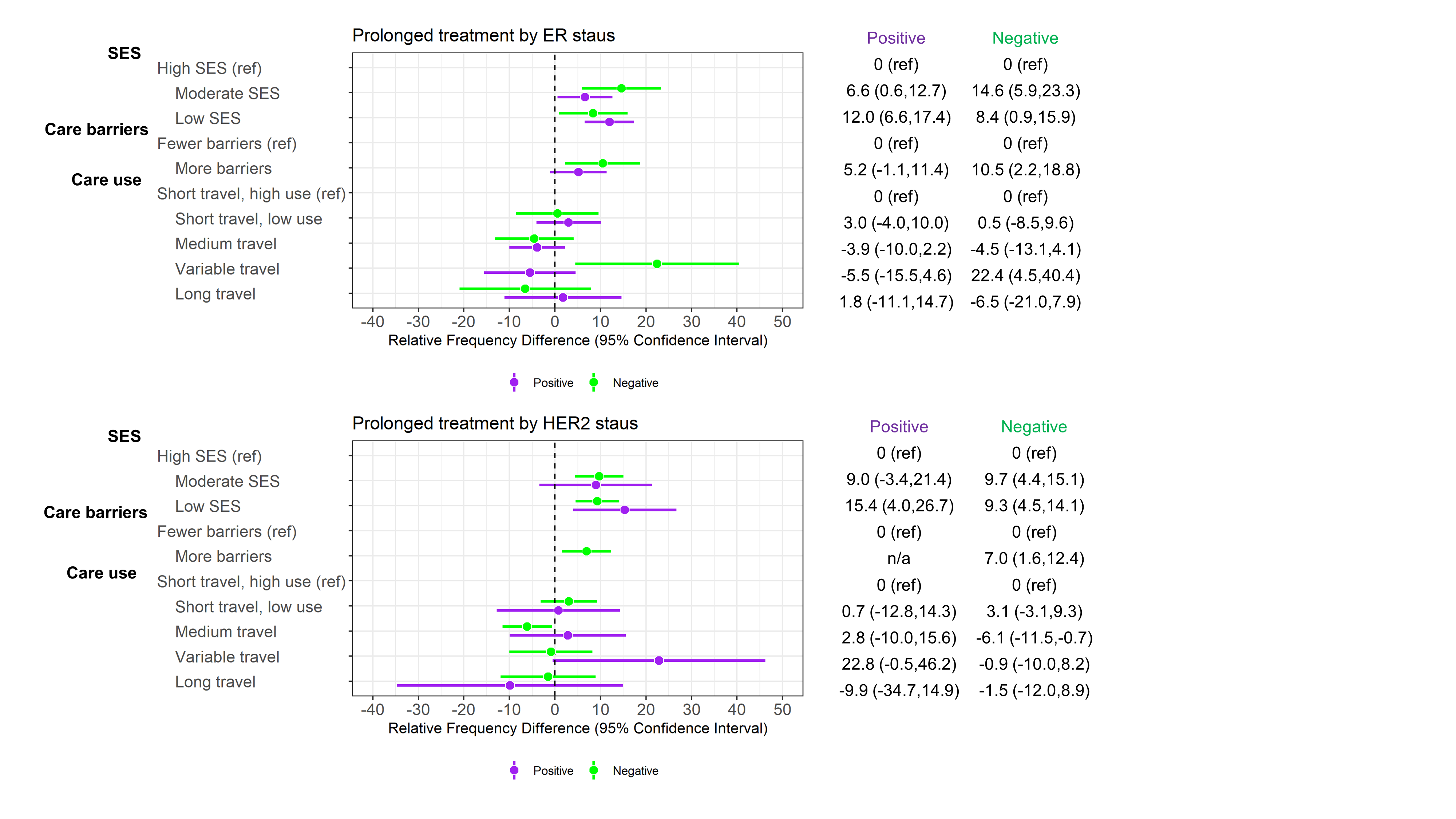

Supplement: S3 Fig — Prolonged treatment was assessed in relation to latent variables defined for SES (income, education, country of birth, job type, and marital status), care barriers (insurance, urban/rural status, job loss, self-reported financial barriers to care, self-reported transportation barriers to care), and care use (pre-diagnostic regular care, breast cancer screening, mode of initial cancer detection (mammogram vs. noticed lump), and travel (based on estimated driving time) to diagnosis and surgery). Contrast estimates are relative frequency differences (RFDs) and 95% confidence intervals, which compare frequency of prolonged treatment for a given latent class with the indicated reference group. Results are stratified by (A) ER status and (B) HER2 status; models are adjusted for age, stage, size, and grade at diagnosis. (TIF) [file pmed.1004500.s008.tif]

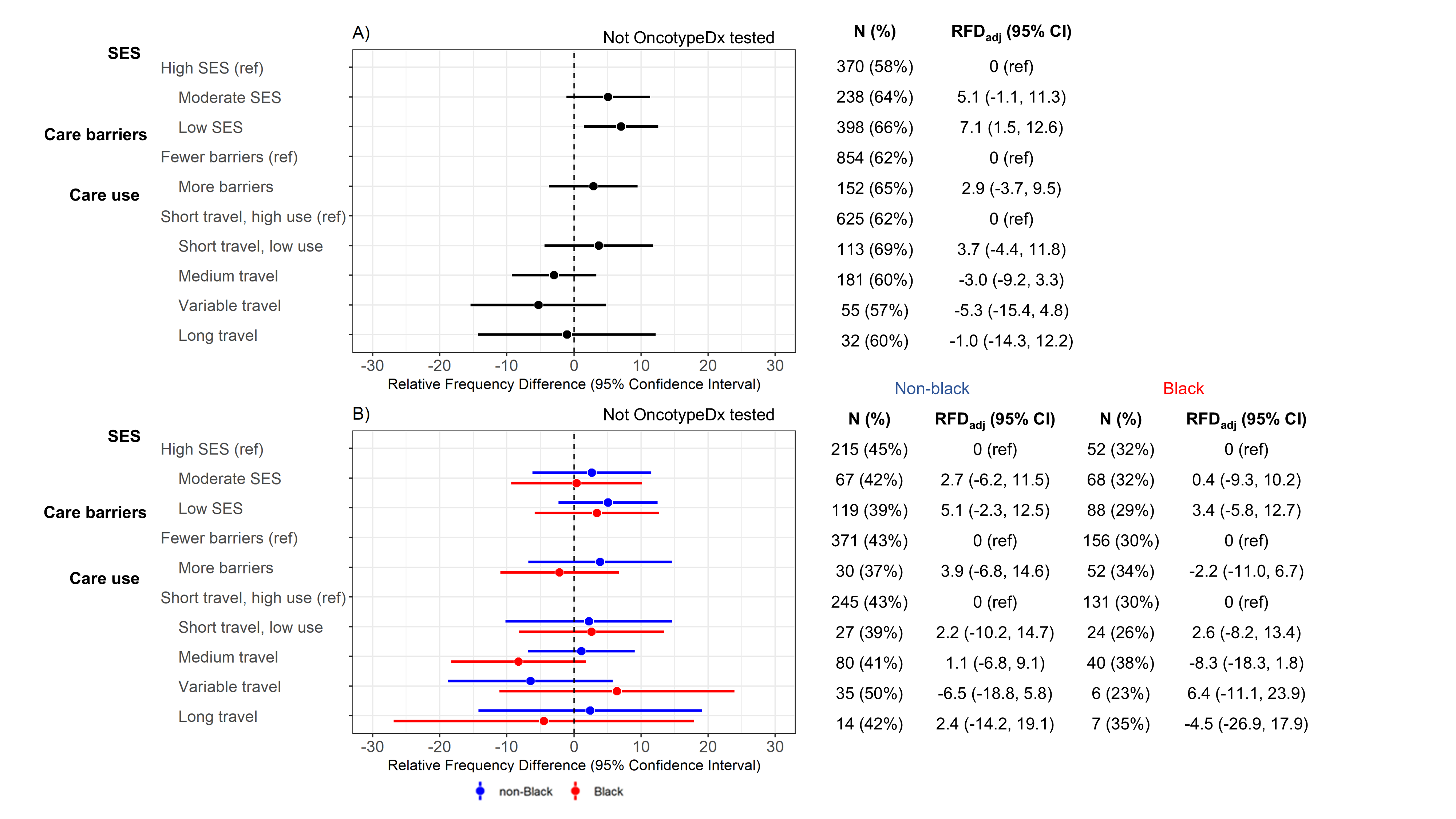

Supplement: S4 Fig — OncotypeDx was assessed among participants with ER+, HER2- disease in relation to latent variables defined for SES (income, education, country of birth, job type, and marital status), care barriers (insurance, urban/rural status, job loss, self-reported financial barriers to care, self-reported transportation barriers to care), and care use (pre-diagnostic regular care, breast cancer screening, mode of initial cancer detection (mammogram vs. noticed lump), and travel (based on estimated driving time) to diagnosis and surgery). Frequency and percentage of delayed diagnosis is reported in “n(%)” columns. Contrast estimates are relative frequency differences (RFDs) and 95% confidence intervals, which compare frequency of being untested for a given latent class with the indicated reference group. Results are presented (A) overall and (B) race-stratified; models are adjusted for age, tumor size, and grade at diagnosis. (TIF) [file pmed.1004500.s009.tif]
