# Supplementary material for: A latent class assessment of healthcare access factors and disparities in breast cancer care timeliness
Source: PLoS Med. 2024 Dec 2;21(12):e1004500. doi: 10.1371/journal.pmed.1004500 (PMC11649116; doi:10.1371/journal.pmed.1004500)
Supplement: Attachment — Submitted filename: PLOSMED_reviewerresponse.docx [file pmed.1004500.s010.docx]

We thank the editor and reviewers for their comments and are grateful for the opportunity to revise our manuscript. Please see our point-by-point responses below. Referenced page and line numbers correspond to the track-change version of the manuscript.

**Comments from the academic editor:**

**Please also looked at the pattern between latent classes (or only generated one set of latent class that included SES, care barriers, and care use), which would provide a richer analyses and go a little bit deeper to bring out the mechanisms of disparities more clearly.**

We agree that examining collective latent class membership across SES, care barriers, and care use, would provide a richer analysis of vulnerability to care delays. The counts and percentages of all possible combinations are presented in Table S4, and the following text was added to the results section (p.16, line 383):

“Overall, there were 30 possible combinations of latent class membership across the domains of SES, care barriers, and care use (Table S4). Each potential combination has at least 3 patients, and 19 out of 30 combinations had at least 30 patients, but there also correlations between domains. For example, low SES patients were more likely to have more care barriers.”

We also generated heat maps (now Figure 5) so that readers can quickly scan and identify which groups had the highest prevalence of delay outcomes. With 30 possible exposure levels, this analysis wasn’t powered for multivariate regression modeling, but we felt that descriptive analysis would still be interesting to the reader. The following paragraph was added to p.18, line 481:

“We then evaluated the outcomes of delayed diagnosis, treatment delay, and prolonged treatment in relation to patterns of latent class membership for all three domains (Figure 5). The prevalence of delayed diagnosis was highest in low travel, low use classes, particularly for patients who also had more care barriers: 46% delayed diagnosis among moderate SES, more barriers, and low travel, low use patients; and 37% among low SES, more barriers, and low travel, low use patients. Delayed treatment was most prevalent among patients with variable or long travel, and had low or moderate SES. Prolonged treatment was associated with lower SES and more barriers groups; the highest prevalence (67%) was observed for the moderate SES, low barriers, and long travel group.”

-----------------------------------------------------------

Comments from the reviewers:

**Reviewer #1: This manuscript describes the result of an analysis of the Carolina Breast Cancer Study cohort of approximately 3000 patients diagnosed 2008-2013. The investigators found racial differences in the care provided to patients with newly diagnosed breast cancer using four outcomes: having a later stage at diagnosis, having a longer time to treatment initiation, having a longer time to treatment completion, and being less likely to have OncotypeDx testing. The analysis also looked at the relationship between three potential latent class predictors (SES, barriers to care, and use of care) and these outcomes, finding that SES and, to a lesser extent care-barriers, were associated with inferior outcomes. Previous studies have demonstrated similar disparities with breast cancer care. This analysis provides interesting findings relative to several clinically relevant outcomes using latent class variables. In fact, the paper is as much about deriving the latent class**

**variables as it is about describing the associations between those variables and the selected outcomes. Several concerns regarding the methods and discussion were noted, as detailed below.**

**An 8-month threshold was used for the treatment duration outcome among chemotherapy recipients. How did the analysis handle patients with HER2-positive and/or stage IV breast cancer? Patients with HER2 positive breast cancer typically receive** **trastuzumab based chemotherapy, where the recommended duration of treatment is at least 12 months. And patients with stage IV breast cancer could receive chemotherapy for >8 months if it is controlling the cancer and not causing bothersome side effects. In either case, treatment would extend beyond the 8-month threshold and could mean that all patients in these groups experienced delayed treatment completion.**

Reviewer #1 raises important points concerning how to define prolonged treatment among later stage and more aggressive cancers. The frequency of prolonged treatment was similar between HER2-positive and HER2-negative patients (46% vs 48%). In our initial submission we included a sensitivity analysis (Now Figure S3) showing that associations were overall quite similar between ER+/ER- and HER2+/HER2- patients. Therefore, we elected to include HER2+ patients in the model.

Although not all stage IV patients had prolonged treatment, the prevalence was still quite high (~80%, much higher than stages I-III). We initially decided to account for this by adjusting for stage, but after further consideration, the study team agrees that the care pathways are likely too different. In the revised manuscript, stage IV patients are now excluded from the prolonged treatment models. Results have been updated for figure 4 and in Table 2.

**The proportion of patients with at least one delay is high - about three quarters. Considering this, it could be interesting to conduct a sensitivity analysis using longer thresholds for delay. Also, it is possible that some outcomes could have been highly correlated with each other. For example, the same patients could have experienced a delayed diagnosis and a prolonged treatment (the predictors for these outcomes were similar). Did the analysis look for correlation between the outcomes?**

We have added a sensitivity analysis (Figure S2) for a 45-day cutoff rather than 30-day cutoff for treatment delay (see response to reviewer # below); results were similar between the analyses.

While some patients experienced multiple delays, there was surprisingly low correlation between the outcomes. For example, the flow diagram (Figure 1) illustrates that about one-third of patients experience treatment delay regardless of whether their diagnosis was late. Similarly, about half of patients in either treatment delay / no treatment delay group would go on to experience prolonged treatment.

The below matrix of tetrachoric coefficients (0=no correlation, 1=perfectly correlated) confirms these variables were not correlated. Thus, we believe a strength of including these outcomes is that they represent distinct markers of delay through the care process.

|  | Delay Diagnosis | Delay  Treat | Prolonged treat |
| --- | --- | --- | --- |
| Delay Diagnosis | 1 | -0.068 | 0.1392 |
| Delay  Treat | -0.068 | 1 | -0.0267 |
| Prolonged treat | 0.1392 | -0.0267 | 1 |

**The approach focuses on a threshold analysis - treatment initiation within 30 days, treatment completion within 2-8 months, etc. This is relatively easy to interpret but makes it hard to understand how much risk is associated with the delay. For example, a two-day delay would have different implications than a 62-day delay. Did the team consider also describing the difference in the median time-to-event?**

We agree time-to-event methods would be ideal for assessing the implications of delay; e.g. for computing relative rates of mortality or recurrence as a dependent variable, with time-to-treatment as an independent variable. For assessing the differences in frequency of delay in association with SES and access factors, however, we believe that a threshold analysis with binary outcomes was more appropriate for the research question, as well as being more interpretable. Thus, we chose cut points supported by literature where any amount of delay is a potential concern

Still, it is true that implications still vary by the extent of delay, and we believe this should be made more clear in the text. We have revised the limitations paragraph (p.24, line 597) to state the following:

“Delay outcomes were also binarized for more interpretable effect estimates, and while avoiding any delay is preferable, there are substantial differences in implications of the length of such delays (e.g., one day vs one month).”

**The analysis defines four outcomes: delayed diagnosis, delayed treatment initiation, delayed treatment completion, and use of oncotypedx. In the text and in Figure 1, the analysis aggregates the first three outcomes into a composite 'any delay' outcome. Consider defining this aggregate outcome in the methods section. Was there a difference in the number of potential delay-events/patient for black vs. non-black patients? Also, compared to the other outcomes assessed by this study, OncotypeDx seems like an outlier. Why should OncotypeDx be included, especially considering that another analysis (reference 61) assessed a similar outcome using the CBCS database.**

We have added a sentence in the exposure assessment to indicate how this was defined (p.11, line 291): “Composite outcomes for delayed diagnosis, delayed treatment, and prolonged treatment were also generated among patients who were eligible for all three outcomes. Patients were classified as having 0,1,2 or all 3 of these outcomes; the relative frequency of each group was presented in Sankey flow diagrams.”

There was some difference by race: 75% of Black patients at least 1 delay, compared to 68% of non-Black patients

Given the continued need to improve equity and uptake of the OncotypeDx test (and ensure patients are not being overtreated when they would not benefit from chemotherapy) we it was of high public health and policy relevance to evaluate OncotypeDx with respect to the healthcare access factors. In previous work from our group, OncotypeDx receipt was evaluated in relation to individual factors, but not latent classes of access.

However, we also agree this outcome is conceptually different from the rest – it is not a delay in a strict sense. Thus, we have decided to move these analyses to the supplementary material to avoid confusion, and make room for the new Figure 5 (see our response to the editor)

**Other potential limitations include that the study only included patients from North Carolina and that all patients were diagnosed 10+ years ago.**

The discussion (p. 25, line 610) has been revised to state these limitations: “Finally, generalizability may be limited by patient geography (North Carolina, USA) and time of diagnosis (2008-2013), and experiences of delay may differ in countries with different health system organization (e.g., countries with universal public insurance).

**The title, "The heterogeneous impact of healthcare access factors throughout the breast cancer care continuum," is confusing and potentially misleading. The analysis includes four outcomes, all focusing on the initial diagnosis/treatment phase of care. No outcomes address other aspects of the care continuum (e.g., survivorship, end of life). If 'throughout the care continuum' is intended to refer to all stages of breast cancer, a different description could be more accurate. The title describes a heterogeneous impact, but it is not altogether clear what is meant by heterogeneity of effect or that this is the major finding of the paper. The title focuses on healthcare access whereas the paper includes a broad set of potential predictors (i.e., SES, care access, care use) which may or may not impact the accessibility of the healthcare system. And the title does not touch on racial disparities, which is a large focus of the analysis and manuscript.**

We thank Reviewer #1 for the thoughtful assessment of our title, and our team agrees it can be substantially improved. ‘Throughout the care continuum’ was intended to represent multiple time points of cancer care, but since survivorship/end of life care was not considered, we have removed this phrase. We used the phrase ‘heterogenous impact’ to summarize that different sets of factors were associated with each outcome, but we recognize that the meaning is not clear, and have deleted this phrase as well.

The title has been revised as follows, to better capture the paper’s focus on timeliness, disparities, and methodology. We did keep the phrase ‘health care access,’ because we interpret ‘access’ as a broader domain that encompasses multiple latent class domains (SES, care barriers, care use)

New title: “A latent class assessment of healthcare access factors and disparities in breast cancer care timeliness”

Reviewer #2:

**1. This study included the first primary breast cancer diagnosis between 2008 and 2013 from CBCS 3. Do the authors have the background data on the possibility of women with breast cancer who were never diagnosed and subsequently not included in this cohort (the original pool from which samples in this manuscript are drawn)? I imagine this neglected group may be from a very different background than those included in the cohort.**

Reviewer #2 is correct in stating that women with more engagement with the health system are more likely to receive a timely diagnosis, and thus would be more likely to be in the eligible universe of patients that were sampled into the Carolina Breast Cancer Study. There may be differences by other factors as well. For example, invasive lobular carcinoma is one type of breast cancer (10-15% of all breast cancers) that is relatively slow-growing and difficult to detect on a mammogram, and thus may have longer time to diagnosis. Unfortunately, we lack data to estimate the prevalence of undiagnosed breast cancer during the study period.

**2. For the sentence "Given that results did not change when restricting to stage I-III cancer (N=2,886), we elected to include patients of all stages": this statement shows an outcome-oriented approach, I would suggest re-phrasing to using a primary analysis set including all stage patients, plus a sensitivity analysis set including only stage I-III cancer and proving the rational for this sensitivity analysis.**

We recognize the wording of this sentence was misleading. We intended to convey that our intention was to include patients of all stages, and sensitivity analyses were performed to assess if restricting to stage I-III cancers would affect the results.

Given that the prolonged treatment models have been revised to exclude stage IV patients (see response to Reviewer #1), and thus 2 out 4 outcomes are now stage I-III only, we deleted the quoted sentence in the study population paragraph. Instead, we have added the following text (p. 10, line 269) to explain why stage IV patients were included in the delayed treatment models.

“Given the relative underrepresentation of stage IV patients in breast cancer health services research, we elected to include patients of all stages in this analysis. Sensitivity analyses showed that results were consistent when restricting to stage I-III patients (Table S3).”

**3. Moreover, have the authors considered a sensitivity analysis comparing black patients with non-Hispanic White patients (the majority of the non-black patient group)?**

This analysis is now provided in Supplemental Table 3; results were very similar as few non-Black patients were non-white.

**4. SES information was obtained approximately 5 months after their breast cancer diagnosis, can the authors give more details on the time interval from diagnosis to SES information collection? The reason for this is that diagnosis and treatment of breast cancer may potentially change their SES.**

It is true that breast cancer diagnosis/treatment can substantially change some aspects of SES, but most of the variables collected (e.g. education, marital status) are stable or mostly stable. There main exception would be income, but the wording of this question in the survey was designed to assess pre-diagnostic household income (“Last year, what was your family income range, before taxes?”)

In the care barriers category, the transportation/financial barrier questions were similarly worded to ascertain pre-dx experiences. For example, “During the past ten years, was there a time you wanted to see a doctor, but could not because of transportation issues?”

**5. It would be very helpful to understand who the patients are by providing the demographic information, including age, tumor size, stage, and grade at diagnosis, by race group.**

This information is now provided in Supplemental Table 1

**6. Table 1: Can the authors provide the breakdown variables of the SES, care barrier and care use factors by race group? If limited by word/table count, this can be shown in the supplementary document.**

This information is now provided in Supplemental Table 2

**7. Figure 1 is very informative and gives an overall picture of the delays. Can the authors add the % of delays (diagnosis stage, initiation delay and prolonged duration) into the figure?**

We appreciate Reviewer #1’s assessment that Figure 1 effectively conveys overall patterns of delays. We have added the prevalence percentages to this figure.

**8. Supplementary Table 1 shows the outcome distribution by group, it is the very first description and modelling results of the study outcome variables, and it should be included in the main manuscript.**

We agree this information should be reported in the main manuscript. These data have been combined with the original Table 1; the new Table 1 is now titled “Distribution of latent class membership and care outcomes by race (N=2,998)”

**9. Figure 2-5: Could the authors add the count and raw percentage of the delays in each SES/Care barrier/use category? It provides the details of the delays in each subgroup, and if a complete data approach is used in the linear-risk regression, the results exclude patients with missing values and thus use fewer patients.**

We have added the count and raw percentage of each delay outcome for Figures 2-5 (now Figures 2-4 and Figure S4)

**10. It seems that by design, the causes are closely correlated with each other, e.g., judging by common sense, one with low SES is probably more likely to have more care barriers and low care use, same for the outcome variables, when one's diagnosis is neglected for some reasons, the same reasons may also play a role in the subsequent treatment. The authors performed their analysis using individual models for one cause on one outcome, separately. Can the authors further indicate how the causes (SES, care barriers and care use) are correlated with each other, and how the outcomes (delay in diagnosis, treatment initiation and prolonged treatment) are correlated with each other? It would be interesting and important to see if the overall effect of a compound variable/cause integrating SES, care barriers and care use together.**

We agree with Reviewer #2’s assessment that assessing correlations between exposures and outcomes would strengthen the paper. A composite variable of SES, care barriers, is presented in Table S4. We then evaluated delay outcomes in relation to this composite variable, shown in Figure 5. Please the response to the academic editor for more detailed information.

The outcomes themselves were surprisingly not correlated with each other, please see the response to reviewer #1 for the table of correlation coefficients

**Reviewer #3: The article "The heterogenous impact of healthcare access factors throughout the breast cancer care continuum" presents associations between latent measures of access to healthcare with timeliness of diagnosis, initiation and completion of treatment; between access to healthcare and receipt of the Oncotype DX genomic test; and describes the differences in the timeliness of care between black and non-black patients.**

-

**The study presents relevant findings for the academic community and health systems, corroborating findings already described elsewhere in the world, and which bring to the table an important discussion related to disparities in access to health services between black and non-black women with breast cancer.**

-

**Below are considerations about the reviews: the article mentions some variables consolidated and studied in another published study of the Carolina Breast Cancer Study, but it is not clear how these variables could be part of this study without being properly described;**

We believe Reviewer #3 is referring to the screening and regular care variables, which are referred to on p.9 (line 234) in the citation “Construction and validation of these two variables are described in more detail in a previous study.” Preceding this sentence is a concise description of the variables, but we wanted to provide the existing paper to readers who wished to learn more.

We have edited the Care Use factors paragraph on p.9 to emphasize these 2 variables are self-reported, which hopefully adds clarity to these variables

**It does not describe the organization of the North Carolina health system, which would be important to understand, for example, how these barriers are configured and whether they are related to the health system, public policies or the living conditions of these women. What should the North Carolina health system guarantee and does not guarantee? since the health systems may consider these findings informative in designing algorithms for identifying and triaging patients for resource-limited patient navigation programs.**

Reviewer #3 is correct that our findings should be interpreted in the context of the United States healthcare system, which is a complex system of public and private payers/insurers and hospitals. While the Affordable Care Act requires all health plans to adhere to certain coverage requirements, experiences may differ greatly from patient to patient in terms of which services are covered, the out-of-pocket costs, etc. Navigation through cancer care may be different in other high-income counties with different health system organizations, e.g., ones with universal public health insurance.

We feel that most of these policy details are out of scope for an investigation that is epidemiological in nature. However, we have revised the conclusion (p.25, line 620) to emphasize that findings should be interpreted in the context of the US health system:

“Such interventions are particularly important given the complexity of the United States healthcare system and related challenges in access and affordability.”

We also added the following limitation to the preceding paragraph (p.25, line 610): “Finally, generalizability may be limited by patient geography (North Carolina, USA) and time of diagnosis (2008-2013), and experiences of delay may differ in countries with different health system organization (e.g., countries with universal public insurance).

-

**Please include the period of data collection and explain how the data was organized? Where it was exported from?**

We have moved this sentence to the study population paragraph (p.7, line 194) to indicate time of data collection: “Study nurses administered in-home surveys to participants approximately 5 months after their breast cancer diagnosis (2008-2013)”

As indicated in the Data Availability statement on the title page, the data was obtained following permission from the Carolina Breast Cancer Study steering committee and IRB approval at the University of North Carolina – Chapel Hill.

We are unsure as to how to further describe how data are organized, other than the current description on p.7 (line 193) which states that “the present study integrates data from participant surveys, medical records, and pathology reports”

-

**There are differences in the income values defined to consider the socioeconomic level of women in the outcome. It was not clear why different values were used.**

**"SES factors. SES information was obtained from an in-home survey administered to participants approximately 5 months after their breast cancer diagnosis. Each variable was defined as a binary or multinomial categorical variable: household income (<$15,000, $15,000-50,000, or $50,000+"**

**"Therefore, we evaluated each care outcome in relation to household income (<20k, 20-50k, 50k+)"**

We thank reviewer #3 for spotting this error; the second use of this variable was <15k, 15-50k, and 50k+ as well, and we have corrected the description to reflect that.

-

**Figure 1 needs to use the terms homogeneously to prevent interpretation by the reader: "initiation" is "treatment initiation" "completion" or "duration".**

We apologize for this inconsistency, figure 1 and its caption have been revised to ensure consistent use of terms

***       We ask every co-author listed on the manuscript to fill in a contributing author statement, making sure to declare all competing interests. If any of the co-authors have not filled in the statement, we will remind them to do so when the paper is revised. If all statements are not completed in a timely fashion this could hold up the re-review process. If new competing interests are declared later in the revision process, this may also hold up the submission. Should there be a problem getting one of your co-authors to fill in a statement we will be in contact. Please do not add or remove authors without first discussing this with the handling editor. You can see our competing interests policy here:** [**http://journals.plos.org/plosmedicine/s/competing-interests**](http://journals.plos.org/plosmedicine/s/competing-interests)**.**

*       **Please upload any figures associated with your paper as individual TIF or EPS files with 300dpi resolution at resubmission; please read our figure guidelines for more information on our requirements:** [**http://journals.plos.org/plosmedicine/s/figures**](http://journals.plos.org/plosmedicine/s/figures)**. While revising your submission, please upload your figure files to the PACE digital diagnostic tool,** [**https://pacev2.apexcovantage.com/**](https://pacev2.apexcovantage.com/)**. PACE helps ensure that figures meet PLOS requirements. To use PACE, you must first register as a user. Then, login and navigate to the UPLOAD tab, where you will find detailed instructions on how to use the tool. If you encounter any issues or have any questions when using PACE, please email us at** [**PLOSMedicine@plos.org**](mailto:PLOSMedicine@plos.org)**.**

Figures have been prepared as 300 dpi TIF files

***       Please ensure that the paper adheres to the PLOS Data Availability Policy (see** [**http://journals.plos.org/plosmedicine/s/data-availability)**](http://journals.plos.org/plosmedicine/s/data-availability))**, which requires that all data underlying the study's findings be provided in a repository or as Supporting Information. For data residing with a third party, authors are required to provide instructions with contact information (web or email address) for obtaining the data. Please note that a study author cannot be the contact person for the data. PLOS journals do not allow statements supported by "data not shown" or "unpublished results." For such statements, authors must provide supporting data or cite public sources that include it.**

Data is available upon request and approval from the Carolina Breast Cancer Study steering committee, as indicated in the data availability statement on the title page

***       We expect all researchers with submissions to PLOS in which author-generated code underpins the findings in the manuscript to make all author-generated code available without restrictions upon publication of the work. In cases where code is central to the manuscript, we may require the code to be made available as a condition of publication. Authors are responsible for ensuring that the code is reusable and well documented. Please make any custom code available, either as part of your data deposition or as a supplementary file. Please add a sentence to your data availability statement regarding any code used in the study, e.g. "The code used in the analysis is available from Github [URL] and archived in Zenodo [DOI link]" Please review our guidelines at** [**https://journals.plos.org/plosmedicine/s/materials-software-and-code-sharing**](https://journals.plos.org/plosmedicine/s/materials-software-and-code-sharing) **and ensure that your code is shared in a way that follows best practice and facilitates reproducibility and reuse. Because Github depositions can
be readily changed or deleted, we encourage you to make a permanent DOI'd copy (e.g. in Zenodo) and provide the URL.**

Although the data is not publicly available to protect patient privacy, we can deposit code in Github prior to publication


OBSERVATIONAL STUDIES
 ***       Abstract: Please include the study design, population and setting, number of participants, years during which the study took place (enrollment and follow up), length of follow up, and main outcome measures.**

These details have been included in the abstract

***       Please ensure that the study is reported according to the STROBE (or appropriate STOBE extension) guideline (available from:** [**https://www.equator-network.org/reporting-guidelines/strobe)**](https://www.equator-network.org/reporting-guidelines/strobe)) **and include the completed STROBE (or STROBE extension) checklist as Supporting Information. Please add the following statement, or similar, to the Methods: "****This study is reported as per the Strengthening the Reporting of Observational Studies in Epidemiology (STROBE) guideline (S1 Checklist)." When completing the checklist, please use section and paragraph numbers, rather than page numbers.**

This checklist has now been included and referenced in the methods

FORMATTING - GENERAL
***       Abstract: Please structure your abstract using the PLOS Medicine headings (Background, Methods and Findings, Conclusions). Please combine the Methods and Findings sections into one section.**
Abstract formatting has been corrected

***       At this stage, we ask that you include a short, non-technical Author Summary of your research to make findings accessible to a wide audience that includes both scientists and non-scientists. The Author Summary should immediately follow the Abstract in your revised manuscript. This text is subject to editorial change and should be distinct from the scientific abstract. Ideally each sub-heading should contain 2-3 single sentence, concise bullet points containing the most salient points from your study. In the final bullet point of 'What Do These Findings Mean?', please include the main limitations of the study in non-technical language. Please see our author guidelines for more information:** <https://journals.plos.org/plosmedicine/s/revising-your-manuscript#loc-author-summary>.

An Author summary has now been included**,** following the abstract

***       Please express the main results with 95% CIs as well as p values. When reporting p values please report as p<0.001 and where higher as the exact p value p=0.002, for example. Throughout, suggest reporting statistical information as follows to improve clarity for the reader "22% (95% CI [13%,28%]; p</=)". Please be sure to define all numerical values at first use.**

We used the following convention (69% vs 57%; RFD= 12.2%, CI: 7.3,16.7) as we felt estimates were more readable without the use of double brackets, but we are amenable to change reporting style based on journal preferences

***       Please include page numbers and line numbers in the manuscript file. Use continuous line numbers (do not restart the numbering on each page).**
These have been included
***       Please cite the reference numbers in square brackets. Citations should precede punctuation.**
Citations have been converted to square brackets

FIGURES AND TABLES
***       Please provide titles and legends for all figures and tables (including those in Supporting Information files).**
These have been provided
***       Please define all abbreviations used in each figure/table (including those in Supporting Information files).**
Abbreviations are defined
*       **Please consider avoiding the use of red and green in order to make your figure more accessible to those with color blindness.**

We have used red/blue and purple/green color contrasts, which are approved for accessibility to persons with color blindness

SUPPLEMENTARY MATERIAL
***       Please note that supplementary material will be posted as supplied by the authors. Therefore, please amend it according to the relevant comments outlined here.

*       Please cite your Supporting Information as outlined here:** [**https://journals.plos.org/plosmedicine/s/supporting-information**](https://journals.plos.org/plosmedicine/s/supporting-information)
Supplemental information citations have been updated

**REFERENCES
*       PLOS uses the numbered citation (citation-sequence) method and first six authors, et al.

*       Please ensure that journal name abbreviations match those found in the National Center for Biotechnology Information (NCBI) databases (**[**http://www.ncbi.nlm.nih.gov/nlmcatalog/journals**](http://www.ncbi.nlm.nih.gov/nlmcatalog/journals)**), and are appropriately formatted and apitalized.

*       Where website addresses are cited, please include the complete URL and specify the date of access (e.g. [accessed: 12/06/2023]).

*       Please also see** [**https://journals.plos.org/plosmedicine/s/submission-guidelines#loc-references**](https://journals.plos.org/plosmedicine/s/submission-guidelines#loc-references) **for further details on reference formatting**

Reference formatting has been updated
