# Supplementary material for: A latent class assessment of healthcare access factors and disparities in breast cancer care timeliness
Source: PLoS Med. 2024 Dec 2;21(12):e1004500. doi: 10.1371/journal.pmed.1004500 (PMC11649116; doi:10.1371/journal.pmed.1004500)
Supplement: Attachment — Submitted filename: PLOS_Med_Response2.docx [file pmed.1004500.s011.docx]

We are delighted to hear our manuscript has been tentatively accepted, and have made further revisions according to the reviewers’ and editor’s comments. Please see our point-by-point responses below.

**Comments from Reviewers:**

**Reviewer #2: Line 249, the newly added content "Effect estimates were similar in sensitivity analyses with Black 249 and White patients only (N=2,916) (S1 Table)" should be moved to the Results section.
Line 272, the newly added content "Sensitivity analyses showed that results were consistent when restricting to stage I-III patients (S3 Table)" should be moved to the Results section.**

The following short paragraph was added to the results section, following presentation of the main models:

“Sensitivity analyses were performed to assess the potential impact of additional exclusion criteria. Effect estimates were similar in sensitivity analyses with Black and White patients only (N=2,916) (S3 Table). Results for the delayed diagnosis and delayed treatment models were also consistent when restricting to stage I-III patients (N=2,886) (S3 Table).”

**The manuscript in its current version uses both Roman numbers and Arabic numbers for cancer stages, can the authors use one form consistently?**

We now refer to cancer stage with Roman numerals consistently; corrections have been made to the manuscript text, table S1

**For Figures 2-4, It is great that the authors added the N(%), it is important to have them there, because 1). It is often good to have the Ns to understand the distribution; 2) without the N (%), the RFD by subgroup would be very misleading, as the reference groups are different between the two subgroups. Can the authors adjust the title on the right "Non-black" and "Black" so that they are above the columns of both N(%) and RFD?**

We agree this is important information to present, and have re-aligned the columns as indicated by Reviewer #2.

**Figure 5 is an interesting one, the delays (diagnosis/treatment/treatment) do not show any strong pattern across the latent class groups (SES, carer barriers, travel), however, the distribution across the latent class groups themselves shows a strong pattern, that is, only very few people in high SES had barriers (all the grey patches). This might be what Reviewer 1 indicated in the comment "…this paper is as much about deriving the latent class variables as it is about describing the associations between those variables and the selected outcomes." I agree with this.**

We appreciate Reviewer #2’s assessment that Figure 5 added to understanding of the paper. It is true that certain combinations had few patients, which did limit our assessment of overall patterns.

Any attachments provided with reviews can be seen via the following link:

------------------------------------------------------------
Requests from Editors:
 **CODE AVAILABILITY

Thank you for agreeing to depositing code in Github prior to publication. Please add a sentence to your data availability statement (in the online submission form) regarding the code used in the study, e.g. "The code used in the analysis is available from Github [URL]”. Please note that because Github depositions can be readily changed or deleted, we encourage you to make a permanent DOI'd copy (e.g. in Zenodo) and provide the URL.**

We have added a sentence to the submission form to indicate the Github URL where our code is uploaded

FINANCIAL DISCLOSURE

**Please ensure to update the financial disclosure section in the online submission form with the details provided in lines 374-376.**

We are unsure of which details this is referring to; these lines are from the results section. However, we will confirm the financial disclosures are consistent between the title page and online submission form.

ABSTRACT

1) **l.91: Please define ‘ER+’ and ‘HER2-‘ at first use, for example: “evaluated among patients with early stage, ER+ (estrogen receptor), HER2-** **(human epidermal growth factor receptor 2) disease”.**

These abbreviations are now defined in the abstract

**2) l.94: CBCS or CBCS3? Please clarify**.

The full name of the study now reads ‘Carolina Breast Cancer Study (CBCS) Phase 3’, and the abbreviation ‘CBCS’ is now used consistently

**3) l.101ff: Throughout, we suggest reporting statistical information as follows to improve clarity for the reader "5.5% (95% CI [2.4,8.5])". When reporting 95% CIs please separate upper and lower bounds with commas instead of hyphens as the latter can be confused with reporting of negative values. Please revise throughout the entire manuscript.**

Effect estimate reporting throughout the text has been revised to the indicated format

**4) In the last sentence of the Abstract Methods and Findings section, please describe the main limitation(s) of the study's methodology.**

We have added the following sentence to the abstract: “The main limitations of this work were inability to make inferences about causal effects of individual variables that formed the latent classes, reliance on self-reported socioeconomic and healthcare history information, and generalizability outside of North Carolina, USA.”

**5) Please include the important dependent variables that are adjusted for in the analyses, similar to what is reported on lines 312-315.**

This information is now presented at the end of the first paragraph in the methods and findings section.

**6) We suggest explaining in the Abstract that "travel" was measured as a function of time.**

We added the following clause to the second paragraph in the methods and findings: “…in which travel is defined by estimated road driving time.”

AUTHOR SUMMARY

**1) l.119: Please define ‘SES’ at first use.**

We have now defined this term

**2) In the final bullet point of 'What Do These Findings Mean?', please include the main limitations of the study in non-technical language.**

We have added the following bullet point:

“A limitation of composite measures of healthcare access (e.g., SES) is that they do not assess the effect of specific factors (e.g., income) and further research is needed to understand which factors should be prioritized for intervention planning”

INTRODUCTION
 **1) l.150: Please define ‘U.S.’ at first use.**

United States of America (USA) is now defined consistently

**2) l.161, please change to “Black women and women with low-SES”. Please note that PLOS Medicine prefers the use of patient-centered language.**
We have revised this sentence accordingly and have examined the rest of the paper for patient-centered language

METHODS AND RESULTS

**1) l.182: Please define ’USA’ at first use and ensure to use a consistent format (earlier you used U.S.). Please revise throughout.**

We now use USA throughout.
 **2) l.191: Please provide the approval number if one was obtained.**

We now indicate (Reference ID 413140) for the IRB approval

**3) l.217: When reporting age, please add a unit, such as ‘years’ (age 40 years). Please revise throughout.**

This has been corrected
 **4) ll.239-240: “Effect estimates were similar in sensitivity analyses with Black and White patients only (N=2,916) (S1 Table).” – Do you mean clinical characteristics were similar between Black and White patients? The n-number also does not match the number in S1 Table (n=2998). Please check and revise.**

Our apologies, this citation was supposed to be S3 Table. This has been corrected (and moved to the results section per comments from Reviewer #2)
 **5) l.241: Please define ‘AJCC’.**

American Joint Committee on Cancer (AJCC) has been defined

**6) l.244: You state that “progesterone receptor (PR) status” was obtained from pathology reports, but it is not reported within the manuscript. Please clarify.**

PR was not used in this analysis and this has now been deleted

**7) l.257: Please define ‘HR’ at first use.**

Hazard ratio is now defined

**8) l.260: “stage IV” – please see the reviewer comment regarding Arabic and Roman numerals and revise accordingly.**
We now use Roman numerals for all references to cancer stage

**9) l.266, please change to: “****that time greater than 38 weeks”**
This has been corrected

**10) l.276: Please note that you alternate between using ‘n’ and ‘N’ when reporting total numbers. Please revise using a consistent format.**

We intended to use ‘N’ for counts; Figures 2-4 have been revised

**11) ll.330-331, we suggest specifying as follows: “****Clinical and demographic characteristics, including details on care barriers and care use, of the study population are reported in S1 and S2 Tables.”**
This sentence has been revised as “Clinical and demographic characteristics, including details on SES, care barriers, and care use of the study population are reported in S1 and S2 Tables”

**12) l.332: For better comprehension, we suggest repeating the total n-number here. For example: “Overall out of 2,998 CBCS participants, 18% had a delayed diagnosis,…”**
The beginning of this sentence now reads “Among the total 2,998 CBCS participants,…”

**13) Table 1: We suggest adding the total n-number for each group (Overall, non-Black, Black) at the top of the table. Also, please add a definition for ‘travel’ below the table, i.e. time versus distance, what Oncotype DX is, and define high, moderate and low SES briefly below the table.**
The total Ns have been added. The Table caption now includes definitions for the SES, care barriers, and care use latent classes:

“Latent variables were defined for SES (income, education, country of birth, job type, and marital status), care barriers (insurance, urban/rural status, job loss, self-reported financial barriers to care, self-reported transportation barriers to care), and care use (pre-diagnostic regular care, breast cancer screening, mode of initial cancer detection (mammogram vs noticed lump), and travel (based on estimated driving time) to diagnosis and surgery)”

The caption also now includes a definition of OncotypeDx (also ‘OncotypeDX’ was revised to ‘OncotypeDx’ for consistency

**14) Figure 1: Please add the total n-number to each group in the figure.**
Each box now includes both the total number and percentage

**15) ll.368-369: “Black women more frequently were classified as having more barriers (24% v 10%; RFD= 13.8%, CI: 11.2,16.5). – compared to? Please clarify.**

We now state “Black women more frequently were classified as having more barriers compared to non-Black women (24% v 10%; RFD= 13.8%, 95% CI [11.2,16.5])”

**16) Figure 2/3/4/5: Please add a definition for ‘travel’ below the table, i.e. time versus distance, and briefly define high, moderate and low SES as well as what ‘barriers’ entails.**

The captions for Figs 2-5 (as well as S2-S4 Figs) now include the following (also used in Table 1):

“Latent variables were defined for SES (income, education, country of birth, job type, and marital status), care barriers (insurance, urban/rural status, job loss, self-reported financial barriers to care, self-reported transportation barriers to care), and care use (pre-diagnostic regular care, breast cancer screening, mode of initial cancer detection (mammogram vs noticed lump), and travel (based on estimated driving time) to diagnosis and surgery)”

**17) ll.428-430: “There was also greater frequency of prolonged treatment among participants with more care barriers (54% vs 47%; RFDadj = 7.3%, CI: 2.4, 12.2).” - compared to? Please clarify and revise throughout for clarity.**

This sentence now reads “There was also greater frequency of prolonged treatment among participants with more care barriers compared to few barriers”

**18) ll.449: When reporting percentages, as here "46% delayed diagnosis among moderate SES", please be sure to provide a denominator to help guide the reader. Please revise throughout and add as appropriate.**
This has been revised accordingly, as well as the 2 paragraphs in the ‘latent class membership’ subsection of the results

**19) Figure 5: Please consider avoiding the use of red (particularly in contrast to grey) in order to make your figure more accessible to those with color blindness.**

We thank the editor for bringing this to our attention. Figure 5 now uses a light blue – dark blue color gradient, with suppressed cells ( N<10) shaded white rather than grey. This should improve accessibility.

**20) ll.464-466: Please revise for clarity.**

This sentence now reads “Non-receipt of OncotypeDx was more common among participants with low SES (66% vs 58%; RFDadj = 7.1%, 95% CI [1.5, 12.6]) and moderate SES (64% vs 58%; RFDadj = 5.1%, 95% CI [-1.1, 11.3]) compared to high SES. (S4 Fig)’ **21) Table 2: Please define ‘CI’. Please add a unit for income.**

We have defined CI and added dollar units (also added to S2 Table)

**22) When revising the figures and tables, including supplementary tables and figures, please keep in mind that figures and tables should be self-explanatory on a stand-alone basis, i.e. sufficient detail is required in the descriptions/legends.**

Captions have been revised as requested above, and we also added the supplemental table captions to the manuscript

DISCUSSION
 **Please remove the ‘Conclusion’ subheading from the Discussion.**
This has been removed

REFERENCES

**Where website addresses are cited, please use the word ‘accessed’ when specifying the date of access (e.g. [accessed: 12/06/2024]).**

The word ‘cited’ has been replaced with ‘accessed’

SUPPLEMENTARY MATERIAL

In the published article, supporting information files are accessed only through a hyperlink attached to the captions. For this reason, you must list captions at the end of your manuscript file. You may include a caption within the supporting information file itself, as long as that caption is also provided in the manuscript file. Do not submit a separate caption file.

When SI files are contained with a single file:
Please label the file as ‘S1 Supporting Information’.
Please apply alphabetical labelling to each table and figure contained within the S1 file. For example, ‘Fig A’ to ‘Fig Z’ and ‘Table A’ to ‘Table Z’.
Plain text does not need to be labelled and can just be given a title as necessary. For example, ‘Statistical Analysis Plan’.
Please cite tables/figures as ‘Fig A in S1 Supporting Information’ and/or ‘Table A in S1 Supporting Information’, for example.
Please cite plain text as, ‘Statistical Analysis Plan in S1 Supporting Information’, for example.

When SI files are uploaded as separate files:
Please label tables as ‘S1 Table’ (so on) and figures as ‘S1 Fig’ (and so on).
Any additional documents (protocols/analysis plans etc.) can be labelled as ‘S1 Protocol’, for example. Please cite items as exactly as labelled.

SOCIAL MEDIA

To help us extend the reach of your research, please provide any X (formerly known as Twitter) handle(s) that would be appropriate to tag, including your own, your co-authors’, your institution, funder, or lab. Please enter in the submission form any handles you wish to be included when we post about this paper.

------------------------------------------------------------
General Editorial Requests

1) We ask every co-author listed on the manuscript to fill in a contributing author statement. If any of the co-authors have not filled in the statement, we will remind them to do so when the paper is revised. If all statements are not completed in a timely fashion this could hold up the re-review process. Should there be a problem getting one of your co-authors to fill in a statement we will be in contact. YOU MUST NOT ADD OR REMOVE AUTHORS UNLESS YOU HAVE ALERTED THE EDITOR HANDLING THE MANUSCRIPT TO THE CHANGE AND THEY SPECIFICALLY HAVE AGREED TO IT.

2) Please ensure that the paper adheres to the PLOS Data Availability Policy (see <http://journals.plos.org/plosmedicine/s/data-availability)>, which requires that all data underlying the study's findings be provided in a repository or as Supporting Information. For data residing with a third party, authors are required to provide instructions with contact information for obtaining the data. PLOS journals do not allow statements supported by "data not shown" or "unpublished results." For such statements, authors must provide supporting data or cite public sources that include it.

3) Please review your reference list to ensure that it is complete and correct. If you have cited papers that have been retracted, please include the rationale for doing so in the manuscript text, or remove these references and replace them with relevant current references. Any changes to the reference list should be mentioned in the rebuttal letter that accompanies your revised manuscript

.
